# A conceptual model of the social–ecological system of nature-based solutions in urban environments

**DOI:** 10.1007/s13280-020-01380-2

**Published:** 2020-09-11

**Authors:** Konstantinos Tzoulas, Juanjo Galan, Stephen Venn, Matthew Dennis, Bas Pedroli, Himansu Mishra, Dagmar Haase, Stephan Pauleit, Jari Niemelä, Philip James

**Affiliations:** 1 Department of Natural Sciences, Faculty of Science and Engineering, Manchester Metropolitan University, Chester Street, Manchester, M1 5GD UK; 2 Department of Architecture, Landscape Architecture, School of Arts, Design and Architecture, Aalto University, Otaniementie 14, 02150 Espoo, Finland; 3 Ecosystems and Environment Research Programme, Faculty of Biological and Environmental Sciences, University of Helsinki, P.O. Box 65, Viikinkaari 2a, 00014 Helsinki, Finland; 4 Department of Geography, School of Environment, Education and Development, The University of Manchester, Arthur Lewis G.037, Oxford Road, Manchester, M13 9PL UK; 5 Landscape Architecture and Spatial Planning Group, Wageningen University & Research, P.O. Box 47, 6700 AA Wageningen, The Netherlands; 6 Department of Landscape Architecture, Institute of Agricultural and Environmental Sciences, Estonian University of Life Sciences, Kreutzwaldi 56/3, 51006 Tartu, Estonia; 7 Humboldt University of Berlin, Alfred-Rühl-Haus, Rudower Chaussee 16, 12489 Berlin, Germany; 8 Technical University of Munich, Emil-Ramann-Str. 6, 85354 Freising, Germany; 9 University of Helsinki, Yliopistonkatu 4, Helsinki, 00014 Finland; 10 School of Science, Engineering and Environment, University of Saflord, Peel Building, M5 4WT Salford, UK

**Keywords:** Multifunctionality, Polycentric governance, Relational values, Sustainable urban planning, Transdisciplinarity

## Abstract

This article provides a perspective on nature-based solutions. First, the argument is developed that nature-based solutions integrate social and ecological systems. Then, theoretical considerations relating to relational values, multifunctionality, transdisciplinarity, and polycentric governance are briefly outlined. Finally, a conceptual model of the social–ecological system of nature-based solutions is synthesised and presented. This conceptual model comprehensively defines the social and ecological external and internal systems that make up nature-based solutions, and identifies theoretical considerations that need to be addressed at different stages of their planning and implementation The model bridges the normative gaps of existing nature-based solution frameworks and could be used for consistent, comprehensive, and transferable comparisons internationally. The theoretical considerations addressed in this article inform practitioners, policymakers, and researchers about the essential components of nature-based solutions. The conceptual model can facilitate the identification of social and ecological interconnections within nature-based solutions and the range of stakeholders and disciplines involved.

## Introduction

This perspective article is a reflection by an interdisciplinary team of authors, which develops a series of arguments on nature-based solutions. The authors’ aim is to elucidate nature-based solutions in addressing challenges involving ecological and social systems within the context of urban planning and management. To achieve this, firstly, the argument is developed that nature-based solutions, as other cognate approaches, emphasise the interdependence of social and ecological systems in urban areas. However, the theoretical considerations of this understating are obscure, inconsistently defined, or absent. Secondly, theoretical considerations regarding relational values, multifunctionality, transdisciplinarity, and polycentric governance are briefly outlined in the context of nature-based solutions. Thirdly, the arguments developed throughout this perspective article are synthesised into a conceptual model of the social–ecological system of nature-based solutions.

## Nature-Based Solutions and Linking Social and Ecological Systems in Urban Areas

Since the 1960s, numerous integrative approaches have emerged that link humans and nature in urban areas (Heymans et al. 2019). For example, green infrastructure, urban forestry, and ecosystem services are integrative approaches because they emphasise the coupling of social and ecological factors within the context of urban planning and management (Tzoulas et al. 2007; Escobedo et al. 2018; Heymans et al. 2019). Nature-based solutions were first proposed by the World Bank in 2008 and subsequently introduced into the academic discourse and promoted by, amongst others, the International Union for Conservation of Nature (IUCN 2012) and the European Commission (EC 2015) (Table 1). Normatively nature-based solutions refer to ecosystem interventions that aim at simultaneously addressing ecological, social, and economic challenges (e.g. due to flooding damages and loses; see Scheuer et al. 2012). This aim inevitably involves the direct and indirect coupling of biophysical and social factors at various spatial and temporal scales. For this reason, addressing the complexity of biophysical and social factor components is an intrinsic system characteristic of nature-based solutions. Therefore, conceptualising nature-based solutions as social–ecological systems ought to facilitate the integration of biophysical and social factors and their interrelationships.

**Table 1:** The development and characteristics of nature-based solutions

Period	Formative and normative characteristics	Original source
Pre-2009	The term nature-based solutions is used without definition in a World Bank report of its ecosystem-based adaptation portfolio	WB (2008)
2009–2011	The concept of *ecosystem-based adaptation* is defined and forms the root of the nature-based solutions concept. CBD (2009) and IUCN (2009a) definition: using, restoring, managing, conserving biodiversity and ecosystem services for cost-effective adaptation to climate change and social, economic, cultural co-benefits The concept of nature-based solutions is mentioned by IUCN (2009b) in the context of, and explicitly by MacKinnon and Hickey (2009) to describe, ecosystem-based adaptation	CBD (2009), IUCN (2009a), MacKinnon and Hickey (2009), Dudley et al. (2010), and MacKinnon et al. (2011)
2012–2016	The concept of *nature-based solutions* is defined, differentiated from other concepts, and its core principles formulated. IUCN (2012) definition: nature’s contribution to tackling global challenges of sustainable development (p.1); Explanation: using, restoring, managing, and conserving biodiversity and ecosystem services; Addressing: poverty, disaster risks, climate change, food security, and social and economic development (p1, p24); Theory: systemic trade-offs and synergies acknowledged EC (2015) definition: actions inspired by, supported by, or copied from nature to address societal challenges (p2); Explanation: using, maintaining, and enhancing natural capital; Addressing: green economic growth, competiveness, disaster risks, human well-being, social inclusion, sustainable urbanisation, restoration of degraded ecosystems, climate change adaptation and mitigation, and risk management and resilience (p2, p4, p24); Theory: systemic trade-offs and synergies emphasised Common principles: cost-effective, measurable, replicable, equitable, participatory, innovatively financed, complementary, locally adapted, appropriate scale, increasing resilience, addressing trade-offs, providing multiple co-benefits, integral to policies, site specific, challenge specific, and with good governance; Additional principles^a^: energy efficient, resource efficient, increasing synergies, increasing jobs, increasing labour input, and providing incremental transitions of economic models	IUCN (2012), Balian et al. 2014, Cohen-Shacham et al. (2016) EC 2015, and Maes and Jacobs 2017
Post-2016	The concept *of nature-based solutions* is entering academic discourse and is consolidated, implemented, and evaluated; EC conceptualisation^b^: Brink et al. (2016), Zölch et al. (2017), Escobedo et al. (2018), Potschin et al. (2016), and Eggermont et al. (2015); EC and IUCN conceptualisation^b^: Kabisch et al. (2016), Nesshöver et al. (2017), Lafortezza et al. (2018), Faivre et al. (2017), Cohen-Shacham et al. (2019) IUCN conceptualisation^b^: no studies	

^a^These additional principles are explicitly emphasised only by the EC conceptualisation

^b^All subsequent studies refer to the original sources, shown in the right-hand column

Nature-based solutions could enhance both the planning and management of urban areas by providing opportunities for collaboration between cognate approaches. There is a general assumption that approaches integrating social–ecological systems are mutually compatible. Indeed, six papers have been published with the purpose of clarifying conceptual links and interconnections between nature-based solutions and other integrative approaches (Eggermont et al. 2015; Faivre et al. 2017; Nesshöver et al. 2017; Pauleit et al. 2017; Escobedo et al. 2018; Cohen-Shacham et al. 2019). For this reason, these six publications were selected for an exploratory content analysis designed to answer the question: what are the conceptual links between nature-based solutions and other approaches? The content analysis focussed on the cognate approaches that were most frequently compared and linked to nature-based solutions (i.e. in three or more of the selected publications; Table 2). This focus was narrow enough for the purpose of informing the iterative discussions for this perspective article, but also wide enough for the emergence of meaningful patterns. Explicit words and phrases that were normative for nature-based solutions, and/or compared and made conceptual links with other cognate approaches, were recorded and categorised for analysis of normative contributions to and goals of nature-based solutions, and on their conceptual links to cognate approaches. An iterative discussion between the team of authors (covering the fields of environmental management, landscape architecture, urban ecology, geographical modelling, and landscape planning) tested the reliability of the categorisation and developed the interpretation and representation of the analysis. This exploratory content analysis showed that (a) cognate but variously framed goals are used to differentiate nature-based solutions from other approaches; (b) the explicit conceptual links are inconsistent and mostly broad; and (c) there are still unclear or missing conceptual links between nature-based solutions and other approaches (Table 2). Consequently, there is a lack of consensus regarding the conceptual links between the different integrative approaches.

**Table 2:** Integrative approaches linking social and ecological systems in cities and the conceptual links between them

Integrative approach^(a)^ (mainly refers to)^(b)^	Publication					
	Eggermont et al. (2015)^(1)^	Faivre et al. (2017)^(2)^	Nesshöver et al. (2017)^(3)^	Pauleit et al. (2017)^(3)^	Escobedo et al. (2018)^(4)^	Cohen-Shacham et al. (2019)^(5)^
Nature-based solutions (site or issue-specific interventions)	Biodiversity and well-being	Multiple social challenges	Soc, environ, and econ problems	Multiple social challenges	Human well-being	Soc, well-being, and biodiversity
Ecosystem-based adaptation (site and issue-specific interventions)	Connected to NBS	~	A part of NBS	Roots and subset of NBS	~	An issue-specific NBS
Ecosystem-based mitigation (site and issue-specific interventions)	Connected to NBS	~	A part of NBS	Roots of NBS	~	An issue-specific NBS
Ecosystem approach (integrated management)^(+)^	Connected to NBS	Closely related to NBS	Principles in designing NBS	#	#	Foundation to NBS
Ecosystem services (valuing ecological functions)	~	Operationalised by NBS	Considerations designing NBS	Implementing and designing NBS	An essential function of NBS	Range provided by NBS
Natural capital (accounting monetary values)	Types 2 and 3 NBS as green growth	NBS enhance, use, conserve	Support human needs by NBS	NBS enhance, use, conserve	~	~
Green infrastructure (spatial planning configurations)	Linked to Type 3 NBS	~	Synonymous or similar to NBS	Strategic planning NBS	~	Infrastructure type NBS
Ecological engineering (habitats and species interventions)	Connected to NBS	#	A version of NBS	#	#	Restoration type NBS

(a) only integrative approaches that could be compared across three or more publications are shown, (b) ‘mainly refers to’ here is meant broadly, not specifically to the publications; (+) integrated management of air, water, land, ecology, and people; normative contribution of each publication: (1) a typology of nature-based solutions comprising Type 1 interventions in protected areas, Type 2 interventions in agricultural areas, and Type 3 interventions in urban areas; (2) a research and innovation agenda for nature-based solutions within the European Union funding context; (3) a comparison between nature-based solutions and cognate integrative approaches; (4) a bibliometric evaluation of links between nature-based solutions and cognate integrative approaches; (5) a comparison of the implementation principles for nature-based solutions and cognate integrative approaches; First row: goals that are nature-based solutions aimed at addressing according to each publication; Remaining rows: explicit conceptual links between nature-based solutions and other cognate integrative approaches made in each publication; (~) the integrative approach is mentioned in the publication but it is not clearly linked conceptually to nature-based solutions; (#) the integrative approach is not mentioned in the publication

The interdependence of humans and nature in urban areas is a consistent conceptual link between the integrative approaches shown in Table 2, but the understanding remains axiomatic. Explicitly (Nesshöver et al. 2017; Pauleit et al. 2017; Escobedo et al. 2018) or implicitly (Eggermont et al. 2015; Faivre et al. 2017), three axiomatic conceptions underpin the integrative approaches shown in Table 2. Firstly, nature provides benefits to people. Secondly, people must manage nature to obtain these benefits. Thirdly, it is necessary to strengthen the role of nature in policy-making processes and planning. Furthermore, empirical evidence supports these axioms (e.g. Lafortezza et al. 2018). Hence, the understanding of the interdependence between social and ecological systems in urban areas provides a consistent conceptual link between different integrative approaches. However, the key theoretical considerations of this understanding of nature-based solutions remain obscure, inconsistently defined, or absent. This may undermine the effective planning and implementation of nature-based solutions. The sections that follow briefly discuss some of these key theoretical considerations.

## Theoretical Considerations Relating to the Planning and Implementation of Nature-Based Solutions

The first set of theoretical considerations relates to relational values. Complementary utilitarian and intrinsic values are necessary for framing discussions on nature-based solutions (Eggermont et al. 2015). However, the concept of nature-based solutions has been criticised as a potential form of neoliberal conservation (Fletcher 2012) and for being closely linked with neo-classical economic thinking (Fletcher 2012; Maes and Jacobs 2017). This is because neo-classical economic thinking presents challenges for protecting nature (Kronenberg 2015). Indeed, Escobedo et al. (2018) felt the need to clarify that nature-based solutions, amongst other integrative approaches, are not necessarily about commodification capitalism, or the neo-liberalisation of nature. Diverse, context-specific and individual-specific values and perceived benefits have been defined as relational values (Chan et al. 2016). Relational values are emphasised in the integrative approach of nature’s contributions to people (Díaz et al. 2015; Pascual et al. 2017). Thus, incorporating relational values would help nature-based solutions to be context specific and to avoid the risks of commodification and monetisation (Colding et al. 2020).

The second set of theoretical considerations relates to multifunctionality. All the integrative approaches presented in Table 2 emphasise the multifunctional aspects of nature (Eggermont et al. 2015; Faivre et al. 2017; Nesshöver et al. 2017; Pauleit et al. 2017; Escobedo et al. 2018). Also, there is empirical evidence to suggest that nature-based solutions are effective in providing diverse social and ecological benefits (Faivre et al. 2017; Lafortezza et al. 2018). Furthermore, due to benefit trade-offs and win-wins, nature-based solutions may provide the advantage of promoting policy coherence (Cohen-Shacham et al. 2019). However, this potential advantage needs to be evaluated using empirical evidence and supported by a robust theoretical context. For instance, the theoretical considerations outlined above, or the links between multifunctional benefits and policy coherence, are rarely explicitly or effectively addressed in empirical research (Faivre et al. 2017; Lafortezza et al. 2018). Therefore, the theoretical aspects of nature-based solutions require further development in order to bridge practical gaps and to link benefit trade-offs and win-wins with policy coherence.

The third set of theoretical considerations relates to transdisciplinarity. Projects implementing nature-based solutions provide opportunities for transdisciplinary research. Transdisciplinary research brings together interdisciplinary and multidisciplinary researchers with users and other stakeholders to co-define the problem and then co-design, co-create, and co-manage the solution (Brandt et al. 2013; Nicolescu 2014). Complexity, uncertainty, and transdisciplinarity are explicitly acknowledged as being central to the concept of nature-based solutions (Eggermont et al. 2015; Nesshöver et al. 2017; Pauleit et al. 2017). This acknowledgement means that nature-based solutions require the effective integration of the reductionism, holism, and systems-thinking research paradigms. For example, such diverse disciplines as economics, ecology and sociology, use different research paradigms in identifying, delineating, measuring, and managing a social–ecological system. When different research paradigms are perceived as incompatible or conflicting, transdisciplinary work may be hindered or undermined (Bodin 2017). So, the implementation of nature-based solutions requires frameworks that connect the necessary research paradigms and link conventional disciplines under common umbrellas (e.g. sustainability science, social ecology sciences, and integrated planning).

The fourth set of theoretical considerations relates to polycentric governance; that is to say arrangements that allow multiple, overlapping, semi-autonomous decision-makers to cooperate, compete, and resolve conflicts between each other (Carlisle and Gruby 2019). For example, polycentric governance may be suitable for management of natural resources and commons (Carlisle and Gruby 2019), urban green infrastructure (Buijs et al. 2016), and social–ecological systems (Andersson et al. 2017). This is because managing land use creates the need to integrate cooperative, competing, and conflicting interests of different public, private, and charitable sector decision-makers. When successful, polycentric governance could enhance the adaptive capacity of, develop an appropriate institutional fit for, and reduce redundancy in the resources being managed (Carlisle and Gruby 2019). Hence, the need to integrate cooperative, competing, and conflicting interests in the implementation of nature-based solutions necessitates polycentric governance. In reality, this conception is difficult to achieve and the necessary level of integration difficult to implement.

## A Conceptual Model for Nature-Based Solutions in Cities

Faivre et al. (2017) developed a research agenda that links nature-based solutions to the European Union Research and Innovation targets, the international policy context, and to relevant knowledge-based initiatives and repositories. This provides a useful conceptualisation of nature-based solutions within the context of European Union funding (Faivre et al. 2017), though this focus may restrict the conceptualisation’s transferability to other regional contexts. An existing typology of nature-based solutions (Eggermont et al. 2015) and a framework for designing and implementing nature-based solutions (Nesshöver et al. 2017) are transferable but the social and ecological components that they include are broad. Thus, existing frameworks could be improved through the enhancement of transferability, consistency, and comprehensiveness.

**Fig. 1:**
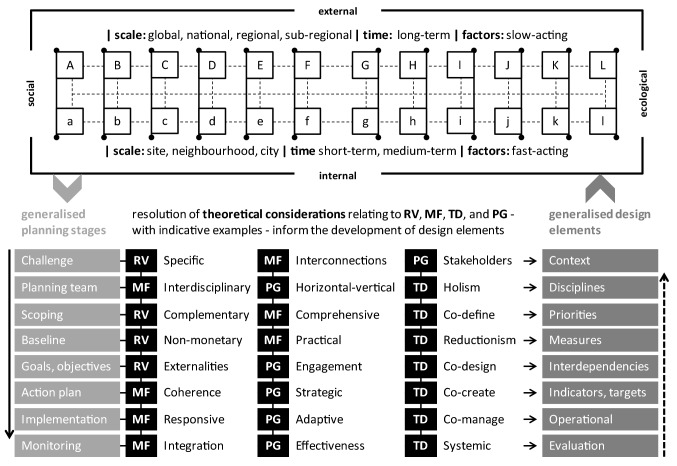
A conceptual model of the social–ecological system of nature-based solutions. This model can be used for conceptualising and for informing planning and implementing nature-based solutions at site level, at the neighbourhood scale, or at the municipal scale. The social–ecological system of a site (upper half of model, outer rectangle) comprises twelve external (**A**–**L**, squares) and twelve internal systems (**a**–**l**, squares). With regard to the site, external systems function slowly at large scales, and internal systems function fast at small, spatial, and temporal scales (upper and lower parts). Social external systems (**A**–**F**) are directly coupled (solid lines, tips on top left of squares) with social internal systems (**a**–**f**) of the site. Ecological external systems (**G**–**L**) are directly coupled (tips on top right of squares) with ecological internal systems (**g**–**l**) of the site. Dynamic and complex interactions, across spatial and temporal scales, indirectly couple (thin dashed lines) all social and ecological, external and internal, systems of the site. The social–ecological system of the site determines (light grey chevron, left) the planning and implementation of a nature-based solution (lower half of the model). The planning and implementation of a nature-based solution includes eight generalised stages (light grey boxes, left) each resulting in different design elements (dark grey boxes, right). Across stages, theoretical considerations emerge: relational values (RV), multifunctionality (MF), transdisciplinarity (TD), and polycentric governance (PG; black boxes, middle). Identifying, framing, and resolving these interrelated theoretical considerations inform (thin arrows, left to right) the design elements of a nature-based solution. Stages and associated design elements are successive (black solid arrow, left), but typically can also be repeated and reviewed for incorporating improvements (black dotted arrow, right). Through continuous iterative reviews, design elements ought to be (dark grey chevron, right) tailored to, and progressively covering the whole of, the social–ecological system of the site

To summarise and synthesise the arguments developed above into a conceptual model, the author team undertook a four-stage iterative, consensus forming discussion of (a) the social and ecological components of nature-based solutions; (b) suitable temporal and spatial scales; (c) the applicability of the four sets of theoretical considerations; and (d) the representation, organisation, and arrangement of the compartments of the model. The outcome of this iterative discussion was the conceptual model depicted in Fig. 1 and Table 3 (Table 3 accompanies Fig. 1). Figure 1 and Table 3 bring together social (A to C), technological (D), political and legal (E), economic (F), ecological (G, H), and environmental (I–L) factors. Thus, the combination of factors creates a comprehensive summary of interconnected social and ecological systems that characterise nature-based solutions.

**Table 3:** Explanations of symbols shown on the conceptual model of the social–ecological system of nature-based solutions in urban environments (Fig. 1)

	External	Internal	Potential indicators for consideration
Social	(A) Demographic (B) Cultural (C) Social (D) Technological (E) Regulatory (F) Financial	(a) People (b) Lifestyles (c) Settings (d) Artificial (e) Operational (f) Economic	Age, sex, ethnicity, education, health, income Diet, recreation, exercise, hobbies, socialising, entertainment, equality Live, work, learn, play, shop, travel, leisure Buildings, transport, utilities, telecoms, digital Ownership, governance, management, maintenance, engagement, policy Capital, revenue, entrepreneurship, returns, accounting, funds, grants, market
Ecological	(G) Biodiversity (H) Land change and N and P flows (I) Freshwater and Oceans (J) Stratospheric O_3_ and aerosols (K) Climate (L) Chemicals	(g) Fauna & flora (h) Soil (i) Water (j) Air (k) Weather (l) Contamination	Patch size, connectivity, disturbance, population management, introductions Compaction, organic matter, contamination, sealing, nutrients, erosion Ground water flows, recharge, inundation, flooding, pollution Smog, heavy metals, particulate matter Urban heat island effect, droughts Heavy metals, endocrine disruptors, bio-accumulation, bio-remediation

This Table accompanies Fig. 1. This Table can be used to identify specific considerations in the planning and implementation of nature-based solutions. Note: External ecological systems are based on planetary boundaries (Hoornweg et al. 2016) and on the ecological model of health promotion (Dustin et al. 2010); internal social systems are based on the settings approach (Poland et al. 2009) and on the determinants of health (Whitehead and Dahlgren 1991); Letters: A to L and a to l reflect the external and internal systems shown in Fig. 1

Figure 1 is applicable to single sites, networks of sites, cities, or larger conurbations. However, in this article, the conceptualisation shown in Fig. 1 is applied to a single site, which is the smallest social–ecological system for a nature-based solution. There are three broad steps in applying Fig. 1 during the planning and implementation of nature-based solutions.

The first step is the conceptualisation of the social–ecological system of a site, which is exemplified as a set of twenty-four external and internal systems (Fig. 1, upper half, and Table 3). External systems function at spatial scales larger than the physical boundaries of the site and are affected by slow-acting processes operating over long timeframes (Fig. 1, upper half, upper part). Internal systems function at spatial scales equal to and smaller than the physical boundaries of the site and are affected by fast-acting processes over medium-to-short timeframes (Fig. 1, upper half, lower part). At each site there are six social and six ecological external systems, each corresponding to respective internal social and ecological systems. For example, the social external system ‘demographic’ (A, Fig. 1 and Table 3) functions at the catchment area of the site, and the social internal system ‘people’ (a, Fig. 1 and Table 3) functions within the site. The ethnicity of the catchment population and the ethnicity of site users may be indicators for these social external and social internal systems, respectively (Table 3). Furthermore, the ecological external system ‘biodiversity’ (G, Fig. 1; Table 3) functions at the catchment scale of the site, and the ecological internal system ‘fauna & flora’ (g, Fig. 1; Table 3) functions within the site. Habitat connectivity at the catchment area and habitat connectivity within the site may be indicators for these ecological external and ecological internal systems, respectively. Therefore, the upper half of the conceptual model can be used to identify and define the social and ecological, internal and external systems of a site.

Social and ecological external and internal systems are based on the models of planetary boundaries (Hoornweg et al. 2016), ecological model of health promotion (Dustin et al. 2010), the settings approach (Poland et al. 2009), and on the determinants of health (Whitehead and Dahlgren 1991). Collectively, this combination of models covers biophysical, biological, and social-economic limits to sustainable development and human well-being, including factors such as physical, psychological, familial, communal, national, international, and global ecological health; and biological, physical, social, economic, and environmental factors (Whitehead and Dahlgren 1991; Poland et al. 2009; Dustin et al. 2010; Hoornweg et al. 2016).

Boundaries between different external and internal systems are fuzzy and there are inevitable overlaps between systems. Figure 1 and Table 3 facilitate navigation through such overlaps. For example, nitrogen and phosphorus are seen as nutrients in the context of soil, but as chemical pollutants in the context of land contamination (Table 3). In another example, how people spend their time is a cultural expression which, in the context of uncodified choices are seen as lifestyle, but in the context of codified choices are seen as operational (Table 3). In this way, overlaps between systems can be used to identify multifunctionality of nature-based solutions. Thus, identifying and dealing with overlaps between systems is one way in which Fig. 1 and Table 3 could be used in the planning and implementation of nature-based solutions.

Figure 1 demonstrates the complexity of interactions between social and ecological external and internal systems. Social external systems are directly coupled with social internal systems (Fig. 1, A–F and a–f, solid lines with tips on top left of squares). Ecological external systems are directly coupled with social internal systems (Fig. 1, G–L and g–l, solid lines with tips on top right of squares). For example, the external system ‘demographic’ is directly coupled with the internal system ‘people’ e.g. changes in ethnicity of catchment population directly affect ethnicity of site users (Fig. 1, A–a). The external system ‘biodiversity’ is directly coupled with the internal system ‘fauna & flora’ e.g. changes in connectivity at the catchment area directly affect connectivity within the site (Fig. 1, G–g). Furthermore, social and ecological internal and external systems are indirectly coupled through complex and dynamic interconnections (Fig. 1, thin dashed lines). For example, ethnicity of the catchment population (social external system) influences habitat connectivity within the site (ecological internal system) via design features and recreational activities targeted at the catchment population. Moreover, the capital and revenue implications of creating design features and providing recreational activities within the site (internal system ‘economic’) are directly coupled with the external system ‘financial’, for example, the availability of central or local government funding (Fig. 1, F–f). Thus, the upper half of the conceptual model can be used to identify complex and inseparable, direct and indirect, couplings between social and ecological, external and internal, systems of a site.

The second step in applying the conceptualisation is using it to identify pertinent theoretical considerations that emerge at different stages of the planning and implementation of nature-based solutions (Fig. 1, lower half of the conceptual model). The planning and implementation process of nature-based solutions can be summarised in eight generalised planning stages (Fig. 1, light grey boxes, left). Each planning stage culminates in the development of a different design element of nature-based solutions (Fig. 1, dark grey boxes, right), after identifying, framing, and resolving relevant theoretical considerations (Fig. 1, thin arrows, left to right). For brevity here, the stages of scoping and monitoring are used to illustrate the application of the conceptual model. At the scoping stage, a survey is undertaken to tailor the nature-based solution to the particular social–ecological system of the site in question. At this stage, priorities have to be set, after considering complementary functions, comprehensive range of systems and stakeholders, and inclusive co-definition processes. These considerations ought to take into account relational values, multifunctionality, and transdisciplinarity, respectively (Fig. 1, black boxes, middle). After implementation, ongoing evaluations are undertaken of social–ecological outcomes, effectiveness of interventions, and systemic feedback mechanisms. These evaluations raise theoretical considerations relating to multifunctionality, polycentric governance, and transdisciplinarity, respectively (Fig. 1, black boxes, middle). Thus, the lower half of the conceptual model can be used to identify theoretical considerations that are pertinent at different stages of the planning and implementation of nature-based solutions.

The final step in applying Fig. 1 is using it as a guide during review processes to develop design elements for the nature-based solution that comprehensively address the social–ecological system of the site. The social–ecological system of the site (Fig. 1, upper half) determines (light grey chevron, left) the planning and implementation of the nature-based solution (Fig. 1, upper half). Planning stages and associated design elements are successive (black solid arrow, left). However, ongoing review processes and monitoring of implementation may reveal the need for amending specific design elements of the nature-based solution. The necessary planning stages are repeated, and design elements are reviewed and amended for incorporating improvements (black dotted arrow, right). Continuous, iterative reviews of the implementation process could improve the design elements, as well as progressively covering the whole of the social–ecological system of the site (dark grey chevron, right). Thus, Fig. 1 can be used to inform monitoring and evaluation processes that can lead to additional design elements that comprehensively address the social–ecological system of the site.

Overall, the upper half of the conceptualisation emphasises the fact that a social–ecological system of a site is complex, dynamic, and operates at multiple temporal and spatial scales. The lower half emphasises that different theoretical considerations emerge at different stages of the planning and implementation of nature-based solutions. The chevron on the left emphasises that the conceptualisation of the social–ecological system of the site determines the planning and implementation of the nature-based solution. The chevron on the right emphasises that the planning and implementation of the nature-based solution in turn defines the conceptualisation of the social–ecological system of the site. The solid and dashed arrows, left and right, respectively, emphasise the circular feedback processes by which the nature-based solution progressively addresses the whole social–ecological system of the site. Thus, this conceptualisation captures the dynamic interactions between the conceptual, theoretical, planning, and implementation challenges of nature-based solutions.

This conceptual model makes three novel contributions to the literature on nature-based solutions. Firstly, the conceptual model brings together the Whitehead–Dahlgren model of health (Whitehead and Dahlgren 1991), the settings approach to health promotion (Poland et al. 2009), the ecological model of health promotion (Dustin et al. 2010), the concept of planetary boundaries (Hoornweg et al. 2016), and urban planning and management concepts. The interdisciplinary synthesis that this conceptual model represents may facilitate its transferability across disciplines. Secondly, the conceptual model comprehensively defines the social and ecological external and internal systems that make up nature-based solutions. The comprehensive definition of social–ecological systems could facilitate the consistent application of nature-based solutions across regions. Finally, this conceptual model emphasises four key theoretical sets of considerations that inform the implementation of nature-based solutions. Thus, the conceptualisation proposed here makes a number of novel contributions to the theoretical understanding of nature-based solutions.

The characteristics of nature-based solutions differ in emphasis and wording between the IUCN (2012) and the EC (2015) (Table 1). The model presented in Fig. 1 and Table 3 helps to bridge the gaps between the normative approaches of the IUCN (2012) and the EC (2015). Figure 1 and Table 3 allow for a comprehensive, consistent, and transferable conceptualisation of nature-based solutions that emphasise social and ecological integration rather than just one or the other. Figure 1 and Table 3 add explicit social and ecological, internal and external system details to the framework for the design and implementation of nature-based solutions presented by Nesshöver et al. (2017), as well as to the typology of nature-based solutions presented by Eggermont et al. (2015). Furthermore, Fig. 1 and Table 3 facilitate the identification of multidisciplinary, interdisciplinary, and transdisciplinary research projects that could be developed in response to the research and innovation agenda on nature-based solutions presented by Faivre et al. (2017). Thus, the model presented here contributes to the advancement of existing frameworks and to the bridging of the normative gaps between them.

Nature-based solutions were compared and linked to nine other cognate approaches, within the publications selected for the exploratory content analysis (Eggermont et al. 2015; Faivre et al. 2017; Nesshöver et al. 2017; Pauleit et al. 2017 Escobedo et al. 2018; Cohen-Shacham et al. 2019). These cognate approaches were catchment system engineering, ecological restoration, ecosystem-based approaches, ecosystem-based disaster risk reduction, forest landscape restoration, natural infrastructure approaches, natural solutions, natural systems agriculture, and urban forestry. The scope of the exploratory content analysis excluded consideration of these nine cognate approaches, because they were linked to nature-based solutions in just one or two of the selected publications (Table 2). A wider range of theoretical considerations than the four considered here would have been revealed had additional cognate approaches been included in the scope of the exploratory content analysis. The exploratory content analysis undertaken to inform this perspective article was inevitably focussed in scope to only frequently compared cognate approaches. Nonetheless, even with such a narrow scope, four theoretical considerations emerged. These indicate the need for further research on conceptual development and diffusion between, and on, ontological, epistemological, and methodological synergies of cognate approaches. Therefore, by focussing on frequently compared cognate approaches, this perspective article has highlighted the need for further research on the normative and theoretical understanding of nature-based solutions.

The breadth of experience and knowledge of the interdisciplinary team of authors allowed iterative discussions on categorisation, analysis, and representation to be informed by a broad range of perspectives (Fig. 1; Table 3). This range of disciplinary perspectives illustrates the need for insights from sustainability science, social ecology sciences, and integrated planning to inform the normative and theoretical understanding of nature-based solutions. Hence, the experience of the team of authors has been central in identifying, articulating, and synthesizing the need for integrating many disciplinary perspectives in the understanding and implementation of nature-based solutions.

A research need arising from the conceptual model presented here (Fig. 1; Table 3) is to test its usability in conceptualising nature-based solutions with academics, policymakers, practitioners, and local community groups. This conceptual model can be used to inform the design, development, management, and/ or monitoring of nature-based solutions in cities. For instance, the potential indicators for consideration (Table 3) can be used by practitioners in the design, implementation, and evaluation of nature-based solutions. Policymakers can use Fig. 1 and Table 3 to draw links between a range of interrelated policy areas. Researchers can use Fig. 1 and Table 3 to develop interdisciplinary, multidisciplinary, and transdisciplinary research. One of the implications of this conceptual model is that a nature-based solution intervention ought to progressively include all of the social and ecological external and internal systems in its design and implementation. Also, the constant, dynamic, and complex interplay between slow (long-term) and fast (short-term) acting processes ought to be explicitly addressed in the design and implementation of nature-based solutions. The theoretical considerations inform practitioners, policymakers, and researchers about the potential design elements of nature-based solutions. Overall, this model has the benefit of conceptualising nature-based solutions in a way that can be transferable at different scales (site, local, municipality) and in different countries around the world. Thus, this conceptual model could be used for consistent conceptualisation, design, and comparison of nature-based solutions internationally.

## Conclusion

The integrative approach of nature-based solutions has the potential to link social and ecological challenges to nature conservation. The social–ecological systems perspective is appropriate for integrating such diverse information and for conceptualising nature-based solutions. In common with other cognate integrative approaches, nature-based solutions emphasise an axiomatic understanding of the importance of nature in urban areas. Figure 1 and Table 3 provide a consistent and transferable way of conceptualising the social–ecological systems that nature-based solutions comprise, as well as outlining key theoretical foundations for consideration at different stages of planning and implementation. Thus, this article makes a contribution to the normative understanding of nature-based solutions and to facilitating their integrated conceptualisation during planning and implementation.

## References

[CR1] Andersson E, Borgström S, McPhearson T, Kabisch N, Korn H, Stadler J (2017). Double insurance in dealing with extremes: Ecological and social factors for making nature-based solutions. Nature-based solutions to climate change adaptation in urban areas, theory and practice of urban sustainability transition.

[CR2] Balian E, Eggermont H, Le Roux X (2014). Outputs of the strategic foresight workshop “nature-based solutions in a BiodivERsA context”.

[CR3] Bodin Ö (2017). Collaborative environmental governance: Achieving collective action in social-ecological systems. Science.

[CR4] Brandt P, Ernst A, Gralla F, Luederitz C, Lang D, Newig J, Reinert F, Abson D, Wehrden H (2013). A review of transdisciplinary research in sustainability science. Ecological Economics.

[CR5] Brink E, Aalders T, Ádám D, Feller R, Henselek Y, Hoffmann A, Ibe K, Matthey-Doret A (2016). Cascades of green: A review of ecosystem-based adaptation in urban areas. Global Environmental Change.

[CR6] Buijs A, Mattijssen T, van der Jagt A, Ambrose-Oji B, Andersson E, Elands B, Steen Møller M (2016). Active citizenship for urban green infrastructure: Fostering the diversity and dynamics of citizen contributions through mosaic governance. Current Opinion in Environmental Sustainability.

[CR7] Carlisle K, Gruby R (2019). Polycentric systems of governance: A theoretical model for the commons. Policy Studies Journal.

[CR8] CBD. 2009. Connecting biodiversity and climate change mitigation and adaptation: Report of the second ad hoc technical expert group on biodiversity and climate change. Technical Series No. 41, Secretariat of the Convention on Biological Diversity, Montreal, Canada. ISBN: 92-9225-134-1.

[CR9] Chan K, Balvanera P, Benessaiah K, Chapman M, Díaz S, Gómez-Baggethun E, Gould R, Hannahs N (2016). Why protect nature? Rethinking values and the environment. PNAS.

[CR10] Cohen-Shacham E, Walters G, Janzen C, Maginnis S (2016). Nature-based solutions to address global societal challenges.

[CR11] Cohen-Shacham E, Andrade A, Dalton J, Dudley N, Jones M, Kumar C, Maginnis S, Maynard S (2019). Core principles for successfully implementing and upscaling nature-based solutions. Environmental Science & Policy.

[CR12] Colding J, Gren Å, Barthel S (2020). The incremental demise of urban green spaces. Land.

[CR13] Díaz S, Demissew S, Carabias J, Joly C, Lonsdale M, Ash N, Larigauderie A, Adhikari JR (2015). The IPBES conceptual framework—Connecting nature and people. Current Opinion in Environmental Sustainability.

[CR14] Dudley, N., S. Stolton, A. Belokurov, L. Krueger, N. Lopoukhine, K. MacKinnon, T. Sandwith, and N. Sekhran (eds.). 2010. Natural solutions: Protected areas helping people cope with climate change, IUCN, WCPA, TNC, UNDP, WCS, The World Bank, WWF, Gland, Switzerland, Washington DC. ISBN: 978-2-88085-308-2.

[CR15] Dustin D, Bricker K, Schwab K (2010). People and nature: Toward an ecological model of health promotion. Leisure Sciences.

[CR16] EC (2015). Towards an EU research and innovation policy agenda for nature-based solutions & re-naturing cities.

[CR17] Eggermont H, Balian E, Azevedo J, Beumer V, Brodin T, Claudet J, Fady B, Grube M (2015). Nature-based solutions: New influence for environmental management and research in Europe. GAIA.

[CR18] Escobedo F, Giannico V, Jim C, Sanesi G, Lafortezza R (2018). Urban forests, ecosystem services, green infrastructure and nature-based solutions: Nexus or evolving metaphors. Urban Forestry and Urban Greening.

[CR19] Faivre N, Fritz M, Freitas T, de Boissezon B, Vandewoestijne S (2017). Nature-based solutions in the EU: Innovating with nature to address social, economic and environmental challenges. Environmental Research.

[CR20] Fletcher R (2012). Orchestrating consent: Post-politics and intensification of nature TM Inc. at the 2012 World Conservation Congress. Conservation and Society.

[CR21] Heymans A, Breadsell J, Morrison GM, Byrne JJ, Eon C (2019). Ecological urban planning and design: A systematic literature review. Sustainability.

[CR22] Hoornweg D, Hosseini M, Kennedy C, Behdadi A (2016). An urban approach to planetary boundaries. Ambio.

[CR23] IUCN (2009). Ecosystem-based adaptation (EbA), position paper, fifteenth session of the conference of the parties to the United Nations Framework Convention on Climate Change (COP15) 7th–18th December, 2009.

[CR24] IUCN (2009). No time to lose—Make full use of nature-based solutions in the post-2012 climate change regime, position paper, fifteenth session of the conference of the parties to the United Nations Framework Convention on Climate Change (COP15) 7th–18th December, 2009.

[CR25] IUCN (2012). The IUCN Programme 2013–2016, adopted by the IUCN World Conservation Congress, September 2012.

[CR26] Kabisch N, Frantzeskaki N, Pauleit S, Naumann S, Davis M, Artmann M, Haase D, Knapp S (2016). Nature-based solutions to climate change mitigation and adaptation in urban areas—Perspectives on indicators, knowledge gaps, barriers and opportunities for action. Ecology and Society.

[CR27] Kronenberg J (2015). Betting against human ingenuity: The perils of the economic valuation of nature’s services. BioScience.

[CR28] Lafortezza R, Chen J, Konijnendijk van den Bosch C, Randrup T (2018). Nature-based solutions for resilient landscapes and cities. Environmental Research.

[CR29] MacKinnon K, Hickey V (2009). Nature-based solutions to climate change. Oryx.

[CR30] MacKinnon K, Dudley N, Sandwith T (2011). Natural solutions: Protected areas helping people to cope with climate change. Oryx.

[CR31] Maes J, Jacobs S (2017). Nature-based solutions for Europe’s sustainable development. Conservation Letters.

[CR32] Nesshöver C, Assmuth T, Irvine K, Rusch G, Waylen K, Delbaere B, Haase D, Jones-Walters L (2017). The science, policy and practice of nature-based solutions: An interdisciplinary perspective. Science Total Environment.

[CR33] Nicolescu B (2014). Methodology of transdisciplinarity. World Futures.

[CR34] Pascual U, Balvanera P, Díaz S, Pataki G, Roth E, Stenseke M, Watson RT, Dessane EB (2017). Valuing nature’s contributions to people: The IPBES approach. Current Opinion in Environmental Sustainability.

[CR35] Pauleit S, Zölch T, Hansen R, Randrup T, van den Bosch CK, Kabisch N, Korn H, Stadler J, Bonn A (2017). Nature-based solutions and climate change—Four shades of green. Nature-based solutions to climate change adaptation in urban areas.

[CR36] Poland B, Krupa G, McCall D (2009). Settings for health promotion: An analytic framework to guide intervention design and implementation. Health Promotion Practice.

[CR37] Potschin, M., C. Kretsch, R. Haines-Young, E. Furman, P. Berry, and F. Baró. 2016. Nature-based solutions. In: *OpenNESS ecosystem services reference book.* ed M. Potschin, K. Jax. EC FP7 Grant Agreement no. 308428.

[CR38] Scheuer S, Haase D, Meyer V, Wong TSW (2012). Spatial explicit multi criteria flood risk—Fundamentals and semantics of multi criteria flood risk assessment. Flood risk and flood management.

[CR39] Tzoulas K, Korpela K, Venn S, Yli-Pelkonen V, Kaźmierczak A, Niemela J, James P (2007). Promoting ecosystem and human health in urban areas using Green Infrastructure: A literature review. Landscape and Urban Planning.

[CR40] WB. 2008. Biodiversity, climate change, and adaptation: Nature-based solutions from the World Bank Portfolio, Report No. 46726, The World Bank, Washington DC, USA.

[CR41] Whitehead M, Dahlgren G (1991). What can be done about inequalities in health?. The Lancet.

[CR42] Zölch T, Henze L, Keilholz P, Pauleit S (2017). Regulating urban surface runoff through nature-based solutions—An assessment at the micro-scale. Environmental Research.

